# Intrathecal magnesium delivery for Mg++-insensitive NMDA receptor activity due to GRIN1 mutation

**DOI:** 10.1186/s13023-023-02756-9

**Published:** 2023-08-03

**Authors:** Sara A. Lewis, Sheetal Shetty, Sean Gamble, Jennifer Heim, Ningning Zhao, Gideon Stitt, Matthew Pankratz, Tara Mangum, Iris Marku, Robert B. Rosenberg, Angus A. Wilfong, Michael C. Fahey, Sukhan Kim, Scott J. Myers, Brian Appavu, Michael C. Kruer

**Affiliations:** 1 Pediatric Movement Disorders Program, Barrow Neurological Institute, Phoenix Children’s Hospital, Phoenix, AZ 85016 USA; 2 Departments of Child Health, Neurology, Cellular & Molecular Medicine, and Program in Genetics, University of Arizona College of Medicine – Phoenix, Phoenix, AZ USA; 3 Valley Anesthesia, Phoenix Children’s Hospital, Phoenix, AZ USA; 4 Department of Nutritional Sciences, University of Arizona, Tucson, AZ USA; 5 Department of Pharmacy & Therapeutics, Phoenix Children’s Hospital, Phoenix, AZ USA; 6 Phoenix Children’s Hospital Biorepository, Phoenix Children’s Hospital, Phoenix, AZ USA; 7 Division of Pediatric Critical Care Medicine, Phoenix Children’s Hospital, Phoenix, AZ USA; 8 Departments of Paediatrics and Neurology, Monash University, Melbourne, VIC Australia; 9 Center for Functional Evaluation of Rare Variants, Emory University, Atlanta, GA USA; 10 Programs in Neuroscience, Molecular & Cellular Biology, and Biomedical Informatics, Arizona State University, Tempe, AZ USA

**Keywords:** **/Terms** epileptic encephalopathy, Cerebral palsy, Dystonia, Neurodevelopmental Disorders, GRIN Disorders, NMDA receptor, Precision Medicine, N-of-1 treatment trial

## Abstract

**Background:**

Mutations in the NMDA receptor are known to disrupt glutamatergic signaling crucial for early neurodevelopment, often leading to severe global developmental delay/intellectual disability, epileptic encephalopathy, and cerebral palsy phenotypes. Both seizures and movement disorders can be highly treatment-refractory.

**Results:**

We describe a targeted ABA n-of-1 treatment trial with intrathecal MgSO_4_, rationally designed based on the electrophysiologic properties of this gain of function mutation in the *GRIN1* NMDA subunit.

**Conclusion:**

Although the invasive nature of the trial necessitated a short-term, non-randomized, unblinded intervention, quantitative longitudinal neurophysiologic monitoring indicated benefit, providing class II evidence in support of intrathecal MgSO_4_ for select forms of GRIN disorders.

**Supplementary Information:**

The online version contains supplementary material available at 10.1186/s13023-023-02756-9.

## Introduction

The N-methyl-D-aspartate (NMDA) ionotropic glutamate receptor is a postsynaptic cation channel that plays a crucial role in excitatory neurotransmission in the brain. Assembled receptors are heterotetramers comprised of two GluN1 (encoded by the *GRIN1* gene) and typically two GluN2 subunits which combine with alternative splicing and post-translational modifications to confer functional specificity. NMDA receptor activation occurs when glutamate binds the GluN2 subunit once glycine has bound the GluN1 subunit. Mg^++^ blocks the cation-permeable pore of the NMDA receptor until the post-synaptic neuronal surface is sufficiently depolarized. At this point, Na^+^ and Ca^++^ influx occurs. Ketamine uncompetitively antagonizes the dizocilpine (MK-801) site within the cation pore region adjacent to the site of Mg^++^ blockade^1^.

GRIN disorders are typically referable to *de novo* mutations in *GRIN1*, *GRIN2A*, *GRIN2B*, *GRIN2C* or *GRIN2D* leading to gain or loss of receptor function [1]. Clinically, GRIN disorders may manifest with developmental and epileptic encephalopathy, intellectual disability, cerebral palsy, autism and/or schizophrenia. Phenotypes may vary considerably depending on the effect of the underlying mutation on NMDA receptor function. Despite increasing knowledge of the molecular basis of GRIN disorders, available treatments have primarily been symptomatic.

We describe a boy with a severe neurodevelopmental disorder (NDD) due to a heterozygous *de novo GRIN1* mutation. In vitro studies indicated that the mutation led to a gain of function effect putatively due to relative insensitivity to Mg^++^-mediated inhibition. We describe our n-of-1 trial experience with both intrathecal magnesium and ketamine as targeted therapies based on this underlying molecular pathophysiology. Magnesium was chosen based on our in vitro findings, while ketamine was also trialed as a clinically-available potentially pore-blocking agent.

## Patient & methods

We report a three year-old boy with a heterozygous *de novo* mutation in *GRIN1* (NM_007327.3 c.1923G > A (p.M641I) (OMIM #614,254)). This variant has been reported in *GRIN1*-associated NDD [2, 3]. The patient was born at term after a healthy pregnancy. Developmental delay was noted at 4 months of age. At age 6 months, infantile spasms emerged with hypsarryhythmia apparent on EEG. Spasms resolved with adrenocorticotropic hormone, leading to transient developmental improvement. Unfortunately, these abilities receded in the ensuing months in association with generalized seizure onset.

Myoclonic and tonic seizures (4–6/day) were frequent despite vagus nerve stimulation and multiple antiepileptic medications. Painful dystonic spasms occurred multiple times per day. At best, interaction remained limited and irritability was prominent while awake (Supplementary Video 1). Additional history and phenotypic findings can be found in Supplemental Clinical Data.

The patient’s (p.M641I) GRIN1 variant was expressed in *Xenopus* oocytes and studied using two electrode voltage clamp (detailed in Supplemental Electrophysiology Methods). Two successive ABA (A = no treatment epoch; B = treatment epoch) n-of-1 treatment trials, using intrathecal magnesium and then ketamine, were designed and implemented based on our in vitro findings. The patient was admitted to the hospital for intensive monitoring after seizure log review indicated stable myoclonic and tonic seizure burden. A peripherally inserted central catheter was placed for blood draws and a C7 intrathecal catheter was placed to serially sample cerebrospinal fluid (CSF) and administer MgSO_4_. Twenty four hours of baseline neurophysiologic and cardiovascular monitoring were collected along with baseline neurological exam and patient video before initiating the n-of-1 trial of intrathecal magnesium. Serial serum and CSF sampling was obtained prior to each successive IT MgSO_4_ dose, accompanied by serial neurologist (supplemented with video recording) and nurse-administered neurological examinations. An overnight washout period followed the conclusion of IT MgSO_4_ administration. Subsequently, a trial of ketamine (5 mg IV, followed by 2 h washout and then slow continuous infusion at a lower rate (0.5 mg/hr; adjusted due to clinical sedation) was administered, again accompanied by continuous cardiovascular and neurophysiologic monitoring.

## Results

**Fig. 1:**
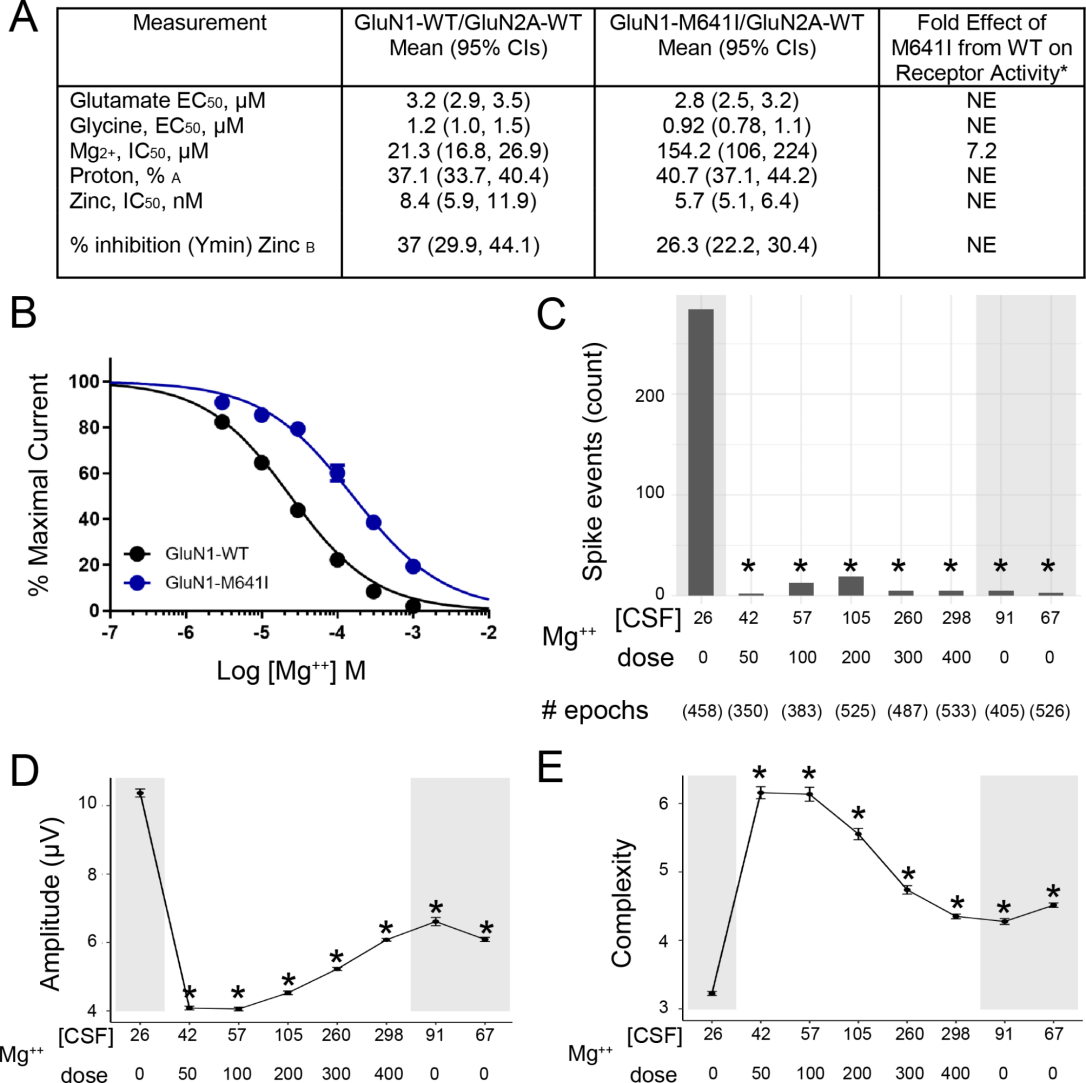
(**A**) In vitro effect on NMDA receptor activity by variant. *Fold effect calculated only when the 95% confidence intervals (CIs) of experimental datasets did not overlap. Positive values indicate a gain of function effect on NMDA receptor activity for the parameter measured. NE = no effect. ^A^ % remaining current measured at pH 6.8 compared to pH 7.6 at maximal L-glutamate and glycine activation; ^B^ % residual current in maximum zinc. Results from n = 10–12 oocytes each. (**B**) A 7 fold reduction in the inhibition sensitivity to endogenous inhibitor Mg^++^ (based on IC_50_ potency) was evident. (**C**) Dose response (spike density, measured as discrete events > 0.0 mV) to escalating doses of intrathecal MgSO_4_ given each hour. Pre-treatment baseline/washout is depicted as gray bar, treatment epochs in white. (**D**) Dose response assessing EEG amplitude pre- and post-treatment; pre-treatment baseline/washout is depicted as gray bar, treatment epochs in white. (**E**) EEG complexity plotted vs. MgSO_4_ dose; pre-treatment baseline/washout is depicted as gray bar, treatment epochs in white. Error bars indicate standard error

The pathogenic variant was found to lead to a relative gain of channel function (Figure 1 A). Intriguingly, agonist and endogenous modulator regulation of the mutant channel was largely preserved, with the notable exception of a marked decrease in sensitivity to voltage-dependent inhibition by Mg^++^, which normally serves to prevent excessive activation (Figure 1 B).

Based on these in vitro findings indicating excessive NMDAR activity in mutant channels, memantine was trialed at age one year but was trialed for only 5 days as seizure burden rose at the time of initiation and the family self-discontinued the medication. Oral and intravenous magnesium were discussed, but considered futile given published data indicating poor blood-brain barrier penetration [4] and little effect on CSF Mg^++^ concentrations [5]. After extensive multidisciplinary discussion and planning, an n-of-1 ABA format trial of IT MgSO_4_ was devised based on perioperative analgesia experience [6] in alignment with consensus recommendations [7] and overseen by the Phoenix Children’s Hospital Institutional Review Board. The total CONsolidated Standards Of Reporting Trials (CONSORT) Extension for N-of-1 Trials (CENT) [8] score was 22/25 (Supplemental Checklist). The Risk of Bias in N-of-1 Trials (RoBiNT) Scale [9] Internal Validity score was 9/14 and the RoBiNT External Validity and Intepretation score was 14/16 for a total score of 23/30 (Supplemental Checklist).

Multimodal cardiovascular and neurophysiologic data (Moberg; Ambler, PA) and video were monitored continuously. An unblinded dose-response trial of IT MgSO_4_ was performed using escalating bolus dosing (Supplemental n-of-1 ABA Trial Methods). Treatment was not randomized given the invasive nature of the intervention. The trial was ended prematurely after the second bolus dose of 400 mg IT MgSO_4_ due to hypotension which responded to intervention.

Intrathecal MgSO_4_ administration led to a marked reduction in spike density and amplitude (Figure 1 C&D) and increase in EEG complexity (Figure 1 E), correlating with a clinical improvement in wakefulness and interaction (Supplemental Video 2). Clinical EEG indicated a reduction in seizures (tonic or behavioral arrest) from 13 to 8 (24 h pre-/post-treatment). Intrathecally administered MgSO_4_ had a CSF magnesium half-life of 31.5 min and concentrations were anticipated to have returned to baseline in 103.8 min.

The day after the IT MgSO_4_ trial, an intravenous (IV) ketamine infusion trial was undertaken. 5 mg ketamine administration led to unacceptable sedation, so 0.5 mg/hr dosing was initiated as a slow infusion and titrated to maximally tolerated dose (0.7 mg/hr; determined by clinical sedation). Ketamine also led to a reduction in spike density (data not shown) consistent with prior reports [10]. The patient was then discharged home where he continued his home medications for several months.

In the outpatient setting, while placement of an intrathecal pump for continuous MgSO_4_ administration was being considered, oral ketamine was initiated and titrated to a dose of 0.5-2 mg/kg/day divided into 4–6 daily doses. This regimen led to complete seizure-freedom for more than 30 days, with periods of improved wakefulness and diminished irritability, although the patient continued to experience intermittent dystonic spasms. Unfortunately, he passed away due to respiratory insufficiency after contacting rhinovirus more than one year after his n-of-1 treatment trial. This outcome was deemed unrelated to his treatment with either ketamine or IT magnesium.

## Discussion

Rare neurogenetic diseases pose significant challenges for affected families, clinicians, and researchers. Nevertheless, advances in our understanding of disease mechanisms are increasingly informing therapeutic development. Our study design utilized CENT Guidelines to maximize rigor and provides level 2 evidence (Oxford Centre for Evidence-Based Medicine) supporting a short-term benefit of IT MgSO_4_. This intervention represented a tailored n-of-1 ABA trial developed directly based on molecular characterization of the patient’s mutation.

Limitations of our study included the non-randomized, unblinded, and short-term nature of our trial. Nevertheless, the majority of study outcome data was automatically captured quantitative neurophysiologic data, and thus not subject to potential observer bias in the same manner that, for example, physician-rated clinical outcome scale scores might be. The use of continuous longitudinal quantitative monitoring in this way (before, during, and after treatment) represents a particular strength of our study. Similar approaches could be utilized to enhance the rigor of subsequent n-of-1 treatment trials.

Although our data indicated a robust neurophysiologic benefit of IT MgSO_4_, it is unclear whether this would translate into clinical improvements of value to the family, such as improved wakefulness, enhanced interaction or developmental milestone attainment. Given the rapid clearance of CSF Mg^++^, it is also unclear if the short-term benefits (neurophysiology and seizure-control) we observed could be sustained over the long run via intrathecal pump-mediated delivery. It is also unclear if supraphysiologic levels of CSF Mg^++^ would lead to additional side effects not observed with short term, bolus dosing. Finally, our study was not designed to assess the efficacy and safety of IT MgSO_4_ in comparison to IV ketamine. All of these considerations affect the generalizability of our findings. Nevertheless, we have provided here first-in-kind evidence that IT MgSO_4_ can be effective in the short-term to ameliorate the neurophysiologic effect of a *GRIN1* gain of function mutation that affects endogenous Mg-mediated inhibition. Our experience also provides instructive data that could be used to further optimize and improve the safety profile of this intervention.

## Conclusions

Our findings indicate a potentially beneficial effect of both ketamine and IT MgSO_4_ for *GRIN1* gain of function mutations. Prior studies using memantine and ketamine [10] have shown potential efficacy in *GRIN2B* gain of function mutations. Despite the neurological disability that typifies GRIN disorders, a Cre-lox mouse model recently indicated that neurodevelopmental deficits associated with *GRIN* mutations can at least partially be recovered in adults [11]. Additional studies will be important to optimize treatments and outcomes for patients with GRIN Disorders.

### Electronic supplementary material

Below is the link to the electronic supplementary material.

Supplementary Material 1

Supplementary Material 2

Supplementary Material 3

Supplementary Material 4

Supplementary Material 5

## Data Availability

Raw data, relevant code, and additional details are available from the corresponding author upon reasonable request.
