# Supplementary material for: Intrathecal magnesium delivery for Mg++-insensitive NMDA receptor activity due to GRIN1 mutation
Source: Orphanet J Rare Dis. 2023 Aug 3;18:225. doi: 10.1186/s13023-023-02756-9 (PMC10398931; doi:10.1186/s13023-023-02756-9)
Supplement: Supplementary file 2 — Supplementary Material 2 [file 13023_2023_2756_MOESM2_ESM.pdf]

Risk of Bias in N-of-1 Trials (RoBiNT) Scale Record Form

This recording form only contains summaries of the rating criteria and should be used in conjunction with the manual

Rater Name: Author & Title:

Internal Validity (IV) Subscale		Score
1<br>**Design with control**	2 Points: At minimum: ABAB with 4 phases; concurrent multiple-baseline design (MBD) with 6 phases, 3 tiers; alternating-treatments design (ATD) with 4 sets of alternating sequences; changing-criterion design (CCD) with 4 steps; for medical N-of-1: 3 x AB pairs<br>1 Point: ABA or 3 phase variant; concurrent MBD with 4-5 phases, 2 tiers; ATD with 3 sets of alternating sequences; CCD with 3 steps; for medical N-of-1: 2 x AB pairs<br>0 Points: AB; AB+follow-up; non-concurrent MBD; ATD with <3 sets of alternating sequences; CCD with <3 steps; nonwithdrawable treatment in ABA	0 **1** 2<br>Where:
2<br>**Randomisation**	2 Points: Randomise: sequence (order) <u>and/or</u> onset (start point) for all phases (see manual for exceptions)<br>1 Point: Restricted randomisation (e.g., participants to blocks of sequences); counterbalancing<br>0 Points: No information; randomisation of other aspects of the study (e.g., stimulus materials)	**0** 1 2<br>Where:
3<br>**Sampling of behaviour**	2 Points: 5 or more data points in <u>every</u> phase with data presented<br>1 Point: at least 3 data points in <u>every</u> phase with data presented<br>0 Points: <3 data points in <u>any</u> phase with data presented	0 1 **2**<br>Where:
4<br>**Blinding of people involved in the intervention**	2 Points: Both participant <u>and</u> practitioner blind to phase of study. If technological intervention used, consult manual<br>1 Point: Participant <u>or</u> practitioner blind to phase. If technological intervention used, consult manual<br>0 Points: <u>Neither</u> participant <u>nor</u> practitioner are blind to phase	**0** 1 2<br>Where:
5<br>**Blinding of assessor(s)**	2 Points: Assessors blind to <u>all</u> phases; use of computer/machine free from human involvement; outcomes self-report <u>and</u> participant is blind<br>1 Point: Independent assessor(s), but not blind to phase<br>0 Points: Practitioner collects/extracts/scores/processes the data; no mention of blinding or independence of assessor(s)	0 1 **2**<br>Where:
6<br>**Interrater agreement**	2 Points: Machine-generated data <u>or</u> data sampled from ≥20% per condition, analysed and reported per condition, with ≥80% agreement (k≥0.6, etc)<br>1 Point: A reasonably objective measure (as defined in the manual) used <u>or</u> agreement is ≥70% (k≥0.4) <u>even if</u> (a) data are not calculated and reported per condition <u>and/or</u> (b) <20% of data is sampled per condition<br>0 Points: Agreement <70% (k<0.4, etc); subjective measure used; consensus ratings alone; inter-rater agreement only reported for a previous study	0 1 **2**<br>Where:
7<br>**Treatment adherence**	2 Points: Machine-delivered intervention free from human implementation <u>or</u> adherence assessed (i) against a clear rating system, (ii) assessor is independent of practitioner/participant, (iii) ≥20% of is data sampled, (iv) resulting in ≥80% adherence<br>1 Point: Adherence meets 2/4 criteria above, <u>and</u> includes (a) assessor independent of practitioner <u>and</u> (b) adherence ≥70%<br>0 Points: Adherence <70%; assessor not independent of practitioner; components only loosely related to adherence	0 1 **2**<br>Where:

© Tate et al. (2015). To be used in conjunction with the RoBiNT manual

External Validity and Interpretation (EVI) Subscale		Score
8<br>**Baseline characteristics**	2 Points: Analysis of baseline characteristics <u>and</u> age, sex, aetiology, severity of condition<br>1 Point: Analysis of baseline characteristics <u>or</u> age, sex, aetiology, severity of condition<br>0 Points: No analysis of baseline conditions <u>or</u> incomplete listing of the four participant characteristics	0 1 2<br>Where:
9<br>**Setting**	2 Points: Description of general location <u>and</u> detailed description of the specific environment<br>1 Point: Description of either general location or specific environment but details are sparse<br>0 Points: <u>Neither</u> general location <u>nor</u> specific environment are described	0 1 2<br>Where:
10<br>**Dependent variable (target behaviour)**	2 Points: Target behaviour is operationally defined in precise terms <u>and</u> the method of measuring it is described<br>1 Point: Target behaviour is operationally defined, but its description and/or method of measurement is not clear and precise<br>0 Points: Target behaviour is not operationally defined	0 1 2<br>Where:
11<br>**Independent variable (therapy/ intervention)**	2 Points: Detailed description of content of the intervention including any equipment/manuals (for medical N-of-1: content of the agents, both active and placebo) <u>and</u> 3 procedural details: number, duration (dosage for medical N-of-1) and frequency of sessions<br>1 Point: General description of content of intervention (and equipment/manuals) <u>and</u> 2/3 procedural details (number, duration/dosage, frequency)<br>0 Points: Intervention described in general terms; only identified as a treatment approach (e.g., "cognitive-behaviour therapy"); <2/3 procedural details	0 1 2<br>Where:
12<br>**Raw data record**	2 Points: Raw data record with a data point for every session/observation period. If ≥10 individual cases, complete raw data record for ≥3 cases<br>1 Point: If ≥10 or more individual cases, complete raw data record for 2 cases, <u>or</u> provision of a data record but data aggregated/averaged across sessions/periods, <u>or</u> provision of data record but a priori decision not to record data for every session (e.g., multiple probe studies)<br>0 Points: No raw data reported; data only reported for selected phases, omitted data	0 1 2<br>Where:
13<br>**Data analysis**	2 Points: Systematic visual analysis with specified protocol, <u>or</u> visual analysis aided by quasi-statistical techniques, <u>or</u> statistical analysis with rationale<br>1 Point: Systematic/aided visual analysis with selection of analytic techniques, <u>or</u> statistical analysis but no rationale, <u>or</u> a priori decision re the level of the target behaviour constituting an empirically derived clinically meaningful change<br>0 Points: Visual inspection without data analysis; analysis not conducted on target behaviour; arbitrary selection of level of target behaviour	0 1 2<br>Where:
14<br>**Replication**	2 Points: 1 original + 3 replications (direct inter-subject or systematic including settings, behaviours, practitioners, intervention)<br>1 Point: 1 original + 1 or 2 replications (inter-subject or systematic)<br>0 Points: No replication	0 1 2<br>Where:
15<br>**Generalisation**	2 Points: Specified generalisation measure is probed in <u>every</u> phase<br>1 Point: Specified generalisation measure is probed in at least pre- and post-treatment phases<br>0 Points: No generalisation measures	0 1 2<br>Where:

Internal Validity subscale: 9 / 14 **External Validity and Interpretation subscale:** 14 / 16 **Total score:** 23 / 30

© Tate et al. (2015). To be used in conjunction with the RoBiNT manual
