# Supplementary material for: Intrathecal magnesium delivery for Mg++-insensitive NMDA receptor activity due to GRIN1 mutation
Source: Orphanet J Rare Dis. 2023 Aug 3;18:225. doi: 10.1186/s13023-023-02756-9 (PMC10398931; doi:10.1186/s13023-023-02756-9)
Supplement: Supplementary file 5 — Supplementary Material 5 [file 13023_2023_2756_MOESM5_ESM.docx]

**HUMAN SUBJECTS CONSIDERATIONS**

The patient’s parents provided their consent to participate in this trial, although it was deemed “not research” (but rather off-label treatment) by the Phoenix Children’s Hospital IRB. The family provided separate written informed consent to have CSF magnesium concentrations determined by ICP-MS via a research protocol and to have their child’s findings published in their present form.

This study was considered level 2 evidence based on Oxford Centre for Evidence-Based Medicine 2011 Levels of Evidence guidelines, downgraded slightly because the intervention in question was not blinded.

**SUPPLEMENTAL CLINICAL DATA**

Motor and language development was minimal. The patient slept much of the day. While awake, he exhibited marked irritability, impaired awareness, limited interaction, and a paucity of volitional movement. Head control was poor, and he did not roll, sit, or reach for toys consistently. Neurological examination revealed generalized hypotonia, a paucity of volitional movement, and diminished visual attention. Episodes of oculogyric crisis were noted. Brain MRI revealed diminished cerebral volume and thin corpus callosum.

Ineffective and/or discontinued anti-epileptic medications at the time of the n=1 trial included topiramate (lack of efficacy), oxcarbazepine (lack of efficacy), phenobarbital (only transient efficacy), fosphenytoin (only transient improvement in seizure burden), zonisamide (limited efficacy), clobazam (limited efficacy and irritability), levetiracetam (irritability), perampanel (limited efficacy and irritability), lacosamide (associated with worsening of seizures), cannabidiol (improved seizure control to discontinued due to worsening dystonia), and memantine (marked increase in seizures; discontinued after only 5 days).

At the time of the n=1 trial, the patient was taking brivaracetam (2 mg/kg/day), perampanel (0.1 mg/kg/day), and valproic acid (45 mg/kg/day). He was on a ketogenic diet (2.5:1 ratio) controlled via G-tube feedings. An implanted vagus nerve stimulator was in place and confirmed to be in good working order.

**Vagus nerve stimulator settings** (at time of trial)

Output current: 1.75 mA

Signal frequency: 30 Hz

Pulse width: 500 μs

On-time: 30 seconds

Off-time: 3 minutes

Autostim Cycle

Output current: 1.875 mA

Pulse width: 500 μs

On-time: 30 seconds

HR sensitivity: 3

Threshold: 20%

Average autostim/day: 180

Magnet Activation

Output current: 2 mA

Pulse width: 500 μs

On-time: 60 seconds

**SUPPLEMENTAL METHODS**

**ELECTROPHYSIOLOGY**

cDNA for the variant receptor GluN1-M641I was generated using QuikChange (Stratagene, Inc). Trafficking studies with this variant have shown it to exhibit normal membrane localization (Xu, et al. 2021).

**mRNA injections** Introduction of the variant into the human GluN1 cDNA (RefSeq NM_007327.3) was verified with Sanger sequencing (Eurofins). The cRNAs for GluN1-M641I or GluN1-WT (control reference allele) were separately combined with human GluN2A-WT cRNA and injected into stage V and VI oocytes and incubated at 16^o^ C for 2–7 days in Barth’s culture medium containing 88 mM NaCl, 2.4 mM NaHCO_3_, 1 mM KCl, 0.33 mM Ca(NO_3_)2, 0.41 mM CaCl_2_, 0.82 mM MgSO_4_, and 5 mM Tris/HCl (pH 7.4). For experiments, oocytes were removed from the incubator and perfused in recording Barth’s solution for two-electrode voltage clamp (TEVC) electrophysiological recordings.

**L-Glutamate dose response studies** The Barth’s solution for these studies contained 90 mM NaCl, 1.0 mM KCl, 0.5 mM BaCl_2_, 10 mM HEPES, 0.01 mM EDTA, adjusted to pH 7.4 (at 23^o^ C) with NaOH. The oocyte membrane potential was clamped at -40 mV. After a steady baseline was obtained, oocytes were perfused with increasing concentrations of L-glutamate (7 concentrations selected from 0.01, 0.03, 0.1, 0.3, 1, 3, 10, 30, 100, 300, or 1000 uM) for 1 min duration each in the continuous presence of 100 uM glycine. Results at each L-glutamate concentration were normalized to the maximum receptor activation levels (defined as 100%) and the EC_50_ values obtained by fitting concentration-response data with Equation 1:

**Equation 1:** Response = 100 / ( ( 1 + EC_50_ / [agonist] ) nH ); where EC_50_ is the agonist concentration that elicited the half maximal response, and nH is the Hill slope.

Statistical comparisons: WT vs variant receptor results were compared using a two-tailed unpaired t-Test (GraphPadPrism 5.0). The log values of the IC_50_ or EC_50_ were used for comparison.

**Glycine dose response studies** The Barth’s solution for these studies was the same as for the L-glutamate studies. Oocyte membrane potential was clamped at -40 mV. After a steady baseline was obtained, oocytes were perfused with increasing concentrations of glycine (7 concentrations selected from 0.01, 0.03, 0.1, 0.3, 1, 3, 10, 30, 100, 300, or 1000 uM) for 1 min duration each in the continuous presence of 100 uM L-glutamate. Results at each glycine concentration were normalized to the maximum receptor activation levels (defined as 100%) and the EC_50_ values obtained by fitting concentration-response data with Equation 1.

**Mg^++^ dose inhibition studies** The Barth’s solution for Mg^++^ studies was identical to that of the L-glutamate dose-response studies except that EDTA was omitted for this condition. The oocyte membrane potential was clamped at -60 mV. After a steady baseline was obtained, oocytes were maximally activated with 100 uM L-glutamate and 100 uM glycine and, in the continuous presence of maximal L-glutamate and glycine, were perfused with increasing concentrations of Mg^++^ (3, 10, 30, 100, 300, and 1000 uM). Results at each Mg^++^ concentration were normalized to maximum receptor activation levels (defined as 100%) and IC_50_ values were obtained by fitting concentration-inhibition data with Equation 2:

**Equation 2:** Response = (100 - minimum) / (1 + ([concentration] / IC_50_)nH ) + minimum; where minimum is the residual percent response in saturating concentration (constrained to > 0) of the experimental compounds, IC_50_ is the concentration of antagonist that causes half maximal inhibition, and nH is the Hill slope.

**pH studies** The Barth’s solution for these studies was identical to that used in the L-glutamate studies. The oocyte membrane potential was clamped at -40 mV. After a steady baseline was obtained in pH 7.6 recording buffer, the oocytes were maximally activated with 100 uM L-glutamate and 100 uM glycine in pH 7.6 buffer. Following a washout period to reestablish baseline, the oocytes were then maximally activated with 100 uM L-glutamate and 100 uM glycine in pH 6.8 buffer. The % current at pH 6.8 was then determined compared to the current at pH 7.6 (defined as 100%).

**Zn^++^ dose inhibition studies** Oocytes expressing recombinant human glutamate receptors were perfused with Barth’s solution identical to that of the L-glutamate dose-response studies except that pH was adjusted to 7.3 (at 23^o^C). The oocyte membrane potential was clamped at -20 mV. After a steady baseline was obtained, oocytes were maximally activated with 50 uM L-glutamate and 50 uM glycine, and then in the continuous presence of maximal glutamate and glycine were perfused with increasing concentrations of Zn^++^ (concentration varies depending on the specific receptor and variant tested). Results at each Zn^++^ concentration are normalized to the maximum receptor activation levels without Zn^++^ (defined as 100%) and IC_50_ values obtained by fitting concentration-inhibition data with Equation 2.

**N-of-1 ABA trial**

**Day 1** The patient was admitted to the pediatric intensive care unit for multimodal continuous neurophysiological monitoring with video EEG. Baseline neurological exam and video were obtained. 24 hours of baseline data was collected. A peripherally inserted central catheter was placed for blood draws and a high cervical (C7) fluoroscopically-guided catheter was placed in order to administer MgSO_4_ and sample CSF and tunneled for stability. Nursing neurological assessments were performed every hour for 3 hours after placement and during MgSO_4_ infusion; every 2 hours at other times. Baseline serum and CSF samples for magnesium determination were collected. 1 ml CSF was collected on ice, stored at -80’C and shipped on dry ice.

**Day 2** A dedicated electronic order set (16.7 mg MgSO_4_/mL;~0.2 mmol elemental Mg^++^ in preservative-free water) was created in preparation for the intrathecal MgSO_4_ administration. Prior studies showed that a total cumulative dose of ~5 mmol (1200 mg cumulative dose) was needed to double CSF concentration in adults receiving continuous cisternal irrigation w/ MgSO_4_ (PMID: 26230471). Given the cumulative risk of infection with an indwelling, repetitively externally-accessed intrathecal catheter, we planned to administer IT MgSO_4_ as a series of escalating slow boluses. We set out to double the MgSO_4_ dose administered (given as a slow push over 5 minutes with normal saline flush) with successive doses every hour as tolerated:

o 1st dose 50 mg MgSO_4_ (0.2 mmol elemental Mg^++^) (3 ml of 16.7 mg/mL MgSO_4_ concentration)

o 2nd dose 100 mg MgSO_4_ (0.4 mmol elemental Mg^++^) (0.5 ml of 200 mg/mL MgSO_4_ concentration)

o 3rd dose 200 mg MgSO_4_ (0.8 mmol elemental Mg^++^) (1 ml of 200 mg/mL MgSO_4_ concentration)

o 4th dose 300 mg MgSO_4_ (1.2 mmol elemental Mg^++^) (1.5 ml of 200 mg/mL MgSO_4_ concentration)

o 5th dose 400 mg MgSO_4_ (1.6 mmol elemental Mg^++^) (2 ml of 200 mg/mL MgSO_4_ concentration)

* Anticipated total cumulative dose at trial completion =

1050 mg MgSO_4_ (4.2 mmol elemental Mg^++^)

Safety measures taken included continuous 5 lead monitoring during the course of the infusion trial. Both clinical response and neurophysiologic variables were assessed while carefully monitoring cardiovascular tolerance. Prior to each bolus administration, serum was sampled via an intravenous peripherally inserted central catheter and CSF was sampled via IT catheter. Serum magnesium goals were <3 mg/dL. As a precaution, team members were briefed on possible adverse effects of hypermagnesemia (at concentrations of 6-12 mg/dL (2.5 to 5 mmol/L) prolongation of PR, widening of QRS, and increased T-wave amplitude on ECG; cardiac arrest may occur when blood magnesium concentration is > 15 mg/dL (6.0 to 7.5 mmol/L).

PRN medications

- IV calcium gluconate 100 mg/kg PRN to counteract effects of hypermagnesemia

- IV furosemide 1 mg/kg plus NS bolus 20 ml/kg as a “flush”

- IV physostigmine 0.5 to 1 mg PRN

The patient tolerated infusions well until the second administration of 400 mg IT MgSO_4_. At this point, he developed hypotension (mean arterial pressure nadir = 35) and was given normal saline boluses (40 ml/kg), calcium chloride (20 mg/kg) and pressor support (phenylephrine infusion) was initiated. He recovered without further events and no additional MgSO_4_ was given.

**Day 3** After the IT MgSO_4_ trial was successfully completed, an IV ketamine infusion trial was undertaken the next day (after an overnight washout based on half-life calculations; see below). Ketamine functions as an uncompetitive NMDAR antagonist, binding to the receptor’s MK-801 site (PMID 23527166). Neurophysiologic data indicated a return to baseline function before the ketamine trial was initiated (data not shown). Ketamine was initiated at a dose of 5 mg (~0.5 mg/kg). Due to excessive sedation, infusion was discontinued. After a 2 hour and 19 minute washout, continuous infusion of ketamine was then restarted at a lower rate (0.5 mg/hr) and serially titrated to maximal tolerated dose (0.7 mg/hr; determined by clinical sedation).

**Day 4** The patient was observed for the morning, and discharged home somnolent but in good health.

**INTRATHECAL MAGNESIUM ELIMINATION RATE**

A single-compartment, first-order model was established based on measureable, non-baseline concentrations to calculate the elimination rate. The point at which concentration returned to baseline was forward extrapolated based on time from previous doses, considered independently from clearance. Predicted peak and trough following each dose were calculated along with theoretical volume of distribution.

**QUANTITATIVE EEG**

Quantitative EEG data was recorded for the patient and spike density, amplitude, complexity and Fast Fourier Transform (FFT) edge calculated using a whole brain region of interest. Spike density, amplitude, complexity and FFT Edge were calculated using Persyst (PMID: 25046981).

**STATISTICAL ANALYSIS**

Planned time-matched epoch data was used to compare pre- and post-treatment values for the four EEG variables mentioned above. Data was analyzed in the context of escalating doses of magnesium and ketamine. Pre- and post-treatment mean values and fold change were further compared using paired t-tests assuming unequal variances, with significance set at p<0.05. For spike density, signal events with voltage > 0 mV were considered a discrete event.
